# The *Schistosoma mansoni* Cytochrome P450 (CYP3050A1) Is Essential for Worm Survival and Egg Development

**DOI:** 10.1371/journal.pntd.0004279

**Published:** 2015-12-29

**Authors:** Peter D. Ziniel, Bhargava Karumudi, Andrew H. Barnard, Ethan M. S. Fisher, Gregory R. J. Thatcher, Larissa M. Podust, David L. Williams

**Affiliations:** 1 Department of Immunology & Microbiology, Rush University Medical Center, Chicago, Illinois, United States of America; 2 Department of Medicinal Chemistry and Pharmacognosy, University of Illinois College of Pharmacy, University of Illinois at Chicago, Chicago, Illinois, United States of America; 3 Skaggs School of Pharmacy and Pharmaceutical Sciences, University of California, San Diego, La Jolla, California, United States of America; McGill University, CANADA

## Abstract

Schistosomiasis affects millions of people in developing countries and is responsible for more than 200,000 deaths annually. Because of toxicity and limited spectrum of activity of alternatives, there is effectively only one drug, praziquantel, available for its treatment. Recent data suggest that drug resistance could soon be a problem. There is therefore the need to identify new drug targets and develop drugs for the treatment of schistosomiasis. Analysis of the *Schistosoma mansoni* genome sequence for proteins involved in detoxification processes found that it encodes a single cytochrome P450 (CYP450) gene. Here we report that the 1452 bp open reading frame has a characteristic heme-binding region in its catalytic domain with a conserved heme ligating cysteine, a hydrophobic leader sequence present as the membrane interacting region, and overall structural conservation. The highest sequence identity to human CYP450s is 22%. Double stranded RNA (dsRNA) silencing of *S*. *mansoni* (*Sm*)CYP450 in schistosomula results in worm death. Treating larval or adult worms with antifungal azole CYP450 inhibitors results in worm death at low micromolar concentrations. In addition, combinations of *Sm*CYP450-specific dsRNA and miconazole show additive schistosomicidal effects supporting the hypothesis that *Sm*CYP450 is the target of miconazole. Treatment of developing *S*. *mansoni* eggs with miconazole results in a dose dependent arrest in embryonic development. Our results indicate that *Sm*CYP450 is essential for worm survival and egg development and validates it as a novel drug target. Preliminary structure-activity relationship suggests that the 1-(2,4-dichlorophenyl)-2-(1H-imidazol-1-yl)ethan-1-ol moiety of miconazole is necessary for activity and that miconazole activity and selectivity could be improved by rational drug design.

## Author Summary

Over 600 million people in endemic countries are at risk of contracting schistosomiasis, which results in over 200,000 deaths each year and significant illness to most people that are infected. There are concerns that the drug widely used for the treatment of schistosomiasis, praziquantel, may be losing efficacy due to evolution of drug resistant worms. Since the disease mainly affects the poor in developing countries, pharmaceutical companies have little interest in developing new drugs and none are currently being tested. In this paper we focus on a novel parasite protein, cytochrome P450, which we propose to be a new drug target. Worms are unusual in having only one cytochrome P450 gene; humans have 57 cytochrome P450 genes. By using reverse genetic and chemical approaches we found that the schistosome cytochrome P450 is essential for worm survival and egg development and, therefore, is an essential and druggable target. Drugs that target fungal cytochrome P450s and are already in use for treating several human diseases were identified as potential hits for further development for schistosomiasis treatment.

## Introduction

Schistosomiasis is a helminthiasis caused by trematode worms of three main schistosome species, *Schistosoma mansoni*, *S*. *haematobium*, and *S*. *japonicum*. The disease is responsible for approximately 280,000 deaths annually and significant morbidity in more than 200 million people [1,2]. Schistosomiasis belongs to a class of neglected tropical diseases whose control has been given limited attention by the pharmaceutic industry because they affect poor people in developing nations. Currently, praziquantel (PZQ) is the only treatment for schistosomiasis [3]. However, studies indicate that PZQ-resistant laboratory strains can be isolated and clinical isolates with increased PZQ resistance have been reported [4]. Therefore, it is a matter of time before resistance fully evolves. In addition, PZQ is much less active against juvenile worms and often results in incomplete cures [5–8] and its mechanism of action, including its biotransformation are not fully understood [3].

Biotransformation pathways play vital roles in providing essential molecules for cell survival and to modify harmful molecules in order to facilitate their elimination. Xenobiotic biotransformation occurs in three phases. Phase I metabolism involves the oxidative, reductive, or hydrolytic transformations of xenobiotics, of which the most important are catalyzed by CYP450 enzymes. In phase II transformation, metabolites undergo conjugation reactions with endogenous compounds such as glutathione, glucuronic acid, amino acids, and sulfate in reactions mainly catalyzed by glutathione *S*-transferases (GSTs), UDP-glucuronosyltransferases, N-acetyltransferases, methyltransferases and sulfotransferases. Phase III transformations utilize membrane-bound transport proteins, which carry modified molecules across membranes for excretion [9]. There has been an extensive study of phase II metabolizing enzymes including the glutathione *S*-transferase family in schistosomes. For example, the main GSTs identified in *S*. *mansoni* have been shown to bind to several commercially available anthelmintics [10] and are currently important vaccine candidates [11]. Recently, a sulfotransferase was implicated in the mechanism and selectivity of action of oxamniquine in schistosomes [12]. In addition, Phase III biotransformation proteins, including the ATP-binding cassette (ABC) transporters, have been identified and their role in praziquantel susceptibility, immunoregulation within the host, parasite egg development and maturation, and translocation of important signaling molecules such as glyco- and phospholipids is being studied [13]. However, very little is known about phase I metabolizing CYP450 enzymes in schistosomes.

CYP450s are heme-containing monooxygenases. In concert with NADPH CYP450 reductases, the heme group of CYP450s serves as a terminal oxidase, i.e., a source of electrons to split molecular oxygen, with one oxygen atom added to the substrate and the other atom accepting reducing equivalents from NADPH to form water [14]. Characterized CYP450 reductase proteins are well conserved and occur as single copy genes in individual organisms. However, the CYP450 proteins are quite diverse, with most organisms having multiple CYP450 genes (Table 1) [9,15]. Analysis of the *S*. *mansoni* genome database has identified only one potential CYP450 gene [16]. In a previous study, extracts of adult *S*. *mansoni* and *S*. *haematobium* were shown to metabolize some typical CYP450 substrates and immunoblotting experiments with an anti-rat CYP450 antibody had cross-reactivity with both *S*. *mansoni* and *S*. *haematobium* homogenates with a specific band at ~50 kDa, well within typical CYP450 molecular weight range [17].

**Table 1:** Comparison of the number of CYP450 and CYP450 reductase genes from different species compiled from Nelson et al. [15]

Organism	CYP450	CYP450 Reductase
*Schistosoma mansoni*	1	1
*Schmidtea mediterranea*	39	1
Human	57	1
*Mus musculus*	103	1
*Gallus gallus*	41	1
*Danio rerio*	81	1
*Drosophila melanogaster*	90	1
*Caenorhabditis elegans*	81	1

In addition to biotransformation activities, CYP450 proteins are involved in the metabolism of many essential endobiotic compounds. Synthesis of membrane sterols, cholesterol and ergosterol depends on CYP450s as does synthesis and degradation of steroid hormones [18,19]. Cellular levels of retinoic acid, the active metabolite of vitamin A, which is essential for embryonic development, postnatal survival, and germ cell development, are regulated and metabolized by several CYP450 proteins [18]. Other CYP450s are involved in the metabolism of prostaglandins, prostacyclins, and leukotrienes [19], all derivatives of fatty acids and important for cell signaling and immune response. In *Caenorhabditis elegans* CYP450 proteins are thought to be involved in meiosis, egg polarization, and egg shell development [20].

**Fig 1:**
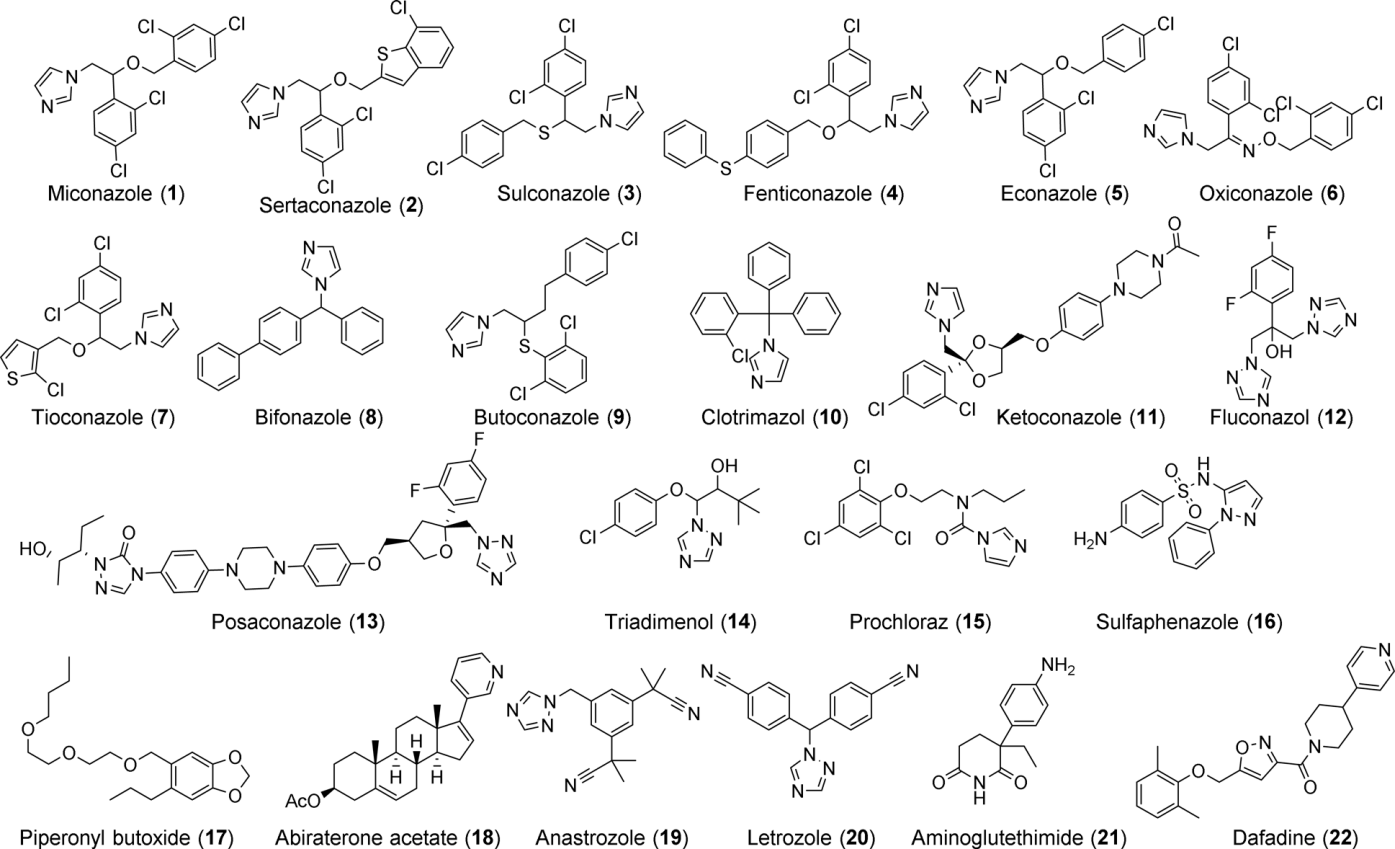
Chemical structures of CYP450 inhibitors used in this study.

In this study, we hypothesize that the single CYP450 gene present in schistosomes is essential for worm survival and that blocking its function would lead to worm death and/or interference in parasite development. We used both genetic and pharmacological approaches to test this hypothesis. Treating larval parasites with *Sm*CYP450-specific double-stranded RNA led to significant decreases in CYP450 mRNA and resulted in worm death. Screening a collection of CYP450 inhibitors (Fig 1) we found that low micromolar concentrations of imidazole antifungal CYP450 inhibitors had schistosomicidal activity against adult and larval worms and blocked embryonic development in the egg. We conclude that *Sm*CYP450 is essential for parasite survival and egg development, and it is proposed as a novel target for antischistosomal drug development, with miconazole analogs as starting points in drug discovery.

## Materials and Methods

### Ethics statement

In all of the experiments involving the use of animals, maintenance and use of these animals were performed in accordance with protocols approved by the Institutional Animal Care and Use Committee (IACUC) at Rush University Medical Center (IACUC number 14–080; DHHS animal welfare assurance number A3120-01). Animals were euthanized with a lethal dose of Nembutal.

### Chemicals and reagents

CYP450 inhibitors (Fig 1) were purchased from Sigma Aldrich (miconazole, clotrimazole, ketoconazole, posaconazole, triadimenol, sertaconazole, bifoconazole, econazole, butoconazole, dafadine, fluconazole), Santa Cruz Biotechnology (piperonyl butoxide, tioconazole, fenticonazole, prochloraz, sulconazole, oxiconazole, anastrozole, letrozole, aminoglutethimide), and Cayman Chemical Company (abiraterone acetate). Sulfaphenazole was synthesized according to published procedures [21,22].

### Experimental organisms

A Puerto Rican strain of *S*. *mansoni* maintained in *Biomphalaria glabrata* snails and the same strain of *S*. *mansoni* maintained in NIH Swiss mice was supplied by the Biomedical Research Institute (Rockville, Maryland, USA). All adult worms, schistosomula, and egg cultures were incubated in Basch’s Media 169 [23]. Basal Medium Eagle was from Life Technologies; glucose and fungizone were from Fisher Scientific; hypoxanthine, serotonin, insulin, hydrocortisone, triiodothyronine were from Sigma Aldrich; MEM vitamins, Schneider’s Drosophila Medium, and gentamicin were from Gibco; HEPES buffer from Mediatech, Inc.; penicillin/streptomycin from Cellgro; and fetal bovine serum was from HyClone Laboratories, Inc.

Cercariae were shed from infected *Biomphalaria glabrata* snails and mechanically transformed to schistosomula as described [24]. To collect liver-stage, juvenile parasites mice were perfused 23 days post infection and to collect adult worms mice were perfused 6–7 weeks after infection with Dulbecco’s modified Eagle’s medium (Gibco) using methods described previously [24]. Live worms were washed thoroughly with DMEM. Eggs were obtained from the livers of the mice 7 weeks post infection. Livers were placed in ice-cold PBS and stored at 4°C overnight and processed the following day as described [24]. Parasite material was stored at -80°C for later use in stage specific *Sm*CYP450 mRNA quantitation.

### Analysis of the *Sm*CYP450 sequence and investigation of sequence variation

The CYP450 open reading frame was amplified from adult mixed cDNA using P450_5' and P450_3' (all primers listed in Table 2) and GoTaq Flexi DNA Polymerase (Promega). PCR product was cloned into pCRII (Invitrogen) and plasmids were purified (Plasmid Mini Kit (QIAGEN) and sequenced at the University of Illinois-Chicago Core Sequencing Center (UIC). Alignment of the obtained open reading frame with the genome sequence was done using the Needleman-Wunsch Global Sequence Alignment Tool (http://blast.ncbi.nlm.nih.gov/Blast.cgi). Prediction of the molecular weight of the encoded protein was done at the Swiss Institute of Bioinformatics Resource Portal (http://web.expasy.org/compute_pi/).

**Table 2:** List of primers used for PCR, RT-PCR and qRT-PCR.

Primer	Sequence
T3	GCTCGAAATTAACCCTCACTAAAGGG
SK	CGCTCTAGAACTAGTGGATC
SL	AACCGTCACGGTTTTACTCTTGTGATTTGTTGCATG
P450_5'	ATGGATACCTTTGAATTTTATG
P450_3'	TTACTTCCATACATCGGTACG
CYP450-R1	CACCATAAGTTGCAACAACG
CYP450-R3	GTTGAGAAGCAGACACATCC
CYP450-revComF2	CTGACTATTGTGTACAGCATA
SmCYP450-For	GTGGACAATTCTGTTGTCTA
SmCYP450-Rev	CTCCAAACTGTACATCTCATC
T7-T3	TAATACGACTCACTATAGGGATTAACCCTCACTAAAGGGA
qPCR-p450-F	TGCTGGTACTGACACCACGTCTTT
qPCR-p450-R	GGAACTACACTAGCCCAACGATGA
qPCR-tubulin-F	CACGAGCAGTTAAGCGTTGCAGAA
qPCR-tubulin-R	TATTTGCCGTGACGAGGGTCACAT
CYP450-interF2	TATGCTGTACACAATAGTCAG
ORF_CYP450 Reverse	TTAAATACTTGTTCTTCTATTTCC
GAPDH_S.mansoni FWD	ATGTTCGTTGTTGGTGTGAATG
GAPDH_S.mansoni REV	TTCCGTTTATGTCTGGAATGA

Internal Coordinate Mechanics (ICM) homology modeling tool (http://www.molsoft.com) [25,26] was used to generate a CYP3050A1 model based on the CYP2C5 (PDB ID 1nr6) template and the structure-superimposition-guided sequence alignments performed using the iterative dynamic programming and superimposition steps implemented in the ICM Homology Modeling module [27]. Alignments were further adjusted manually to preserve integrity of the a-helices and b-sheets, patterns of positive (blue) and negative (red) charges, aromatic (purple) and hydrophobic (green) functionalities, and finally, proline (ochre) and cysteine (yellow) side chains. Global optimization was performed using the Biased Probability Monte Carlo (BPMC) conformational search combined with the electrostatic energy term [28]. Loop search and side chain refinement was conducted for up to 100,000 iterations, which included full energy minimization at each step, to result in a model with satisfactory local strain parameters [29].

To determine if a subset of CYP450 mRNAs was trans-spliced, the *S*. *mansoni* trans-spliced leader sequence was used in PCR with either CYP450-R1 or CYP450-R3 specific internal primers (Table 2). A modified 5’ rapid amplification of cDNA ends (RACE) with Q5 DNA polymerase (New England Biolabs) was done in a nested PCR using an adult cDNA library (kindly provided by Dr. Philip LoVerde) as the template and vector primer T3 + gene-specific CYP450-revComF2 in the first stage and the vector primer SK + gene-specific SmCYP450-Rev (Table 2) for the second stage. The product of the second PCR was cloned into pCR4 (Invitrogen). To determine if the *Sm*CYP450 mRNA is alternatively spliced, the complete ORF was amplified using Q5 DNA polymerase from adult male, adult female, and egg cDNA (synthesized as described below) with P450_5' and P450_3'. PCR products were cloned into pCR4. Plasmid DNAs were isolated (GeneJET Plasmid Miniprep Kit, Thermo Scientific) and sequenced at the UIC sequencing core.

### RNA interference (RNAi)

#### Plasmid Construction

A 566 bp *Sm*CYP450 sequence close to the N-terminal region of the *Sm*CYP450 gene was amplified using SmCYP450-For and SmCYP450-Rev and cloned into a pCRII vector (Invitrogen) according to the manufacturer’s protocol. The sequence was verified through Sanger sequencing at the UIC sequencing core. A new primer (T7-T3) was designed flanking the 566 bp sequence so that it included the full T7 promoter primer followed by part of the T3 primer sequence. PCR was carried out using T7 and T7-T3 primers with Taq DNA polymerase (Thermo Scientific) at 96°C 2 min, followed by 40 cycles at 94°C, 1 min; 48°C, 2 min; 72°C, 1.5 min; then 72°C, 7 min. The PCR product was run on a 1% agarose gel containing ethidium bromide to verify the insert size. The PCR product was cut out from the gel and cleaned with Gel Extraction kit (Qiagen) and the concentration determined.

#### CYP450 dsRNA Synthesis

Both a published method [30] using T7 RNA polymerase (New England BioLabs) and the MEGAclear kit (Life Technologies) were used to synthesize *Sm*CYP450 dsRNA. In the first method, synthesis was carried out in a 100 μl reaction mix using 100 μg/ml BSA (NEB), 500 μM each of rNTPs (NEB), 1 x RNA Pol reaction buffer (40 mM Tris-Cl, 6 mM MgCl_2_, 10 mM dithiothreitol), and 800 units/ml RNase inhibitor (NEB) at 40°C for 4.5 hours. The resultant product was treated with RNAase free DNase I (NEB) at 37°C for 10 min and cleaned using Zymogen DNA-free RNA kit and eluted with DNase/RNase free water, or the DNase I treated samples were precipitated in 75% DEPC treated ethanol and 4 M LiCl and re-suspended in DEPC treated water. The concentration of the cleaned ssRNA was determined using a Nanodrop spectrophotometer. Synthesis using the MEGAclear kit followed manufacturer’s protocols with RNA products cleaned as described above. The RNAs were annealed to form dsRNA by incubating at 75°C, 50°C, and 37°C for 3 min each, and the concentration was determined by Nanodrop spectrophotometry. A negative control dsRNA was synthesized as described above from the *ccdB* and *camR*- bacterial gene insert of pJC53.2 plasmid obtained from Dr. James Collins (UIUC) [30].

#### RNAi Cultures

Freshly prepared schistosomula (300–400) were placed in each well containing 1 ml Basch’s media in a 24-well plate and incubated overnight in a 37°C and 5% CO_2_. The following day *Sm*CYP450 dsRNA or control irrelevant dsRNA was added to each well to a final concentration of 10 or 30 μg/ml. Treatments were done in duplicate. Over several days worms were observed for dead (dark, granular appearance and non-motile) or alive (translucent and motile) as described [31].

### Inhibitor treatment

To determine the activity of CYP450 inhibitors, 10 worm pairs in 5 ml Basch’s media per well in 6-well plates were cultured overnight at 37°C and 5% CO_2_ and the following day CYP450 inhibitors (Fig 1) were added to each well. The media were replenished every 48 hr with fresh media and inhibitors. Dead worms were identified as those that showed no motility when observed for several minutes. For larval worms, 300–400 freshly prepared schistosomula were placed in each well in a 24-well plate containing 1 ml Basch’s Media and incubated overnight at 37°C and 5% CO_2_. The following day compounds were added to each well and the parasites observed for several days without changing the media or adding fresh compounds. Live and dead parasites were classified as before.

To monitor the effects of miconazole on egg development we followed a recently published method [32]. Freshly perfused adult worm pairs were incubated in Basch’s media overnight. The following day worms were removed and miconazole (5 or 10 μM) or an equal volume of DMSO was added to the eggs produced. Eggs were further incubated a total of 72 hr in the presence of miconazole. Each group of treated eggs was then collected and centrifuged (500 x g, 5 min) and the supernatant discarded. The egg pellets were each washed in excess PBS and centrifuged. The eggs were then fixed in 100% methanol at room temperature for 10 min. After removing the methanol the eggs were incubated in DAPI (4’6-diamidino-2-phenylindole) Fluoromount-G (SouthernBiotech) overnight at 4°C for nuclear staining. Images were captured using Zeiss Axiovert Z1 imaging microscope and analyzed with AxioVision software LE (release 4.8.2 SP3, 2013).

### Combined inhibitor and RNA interference

To see if their activities had additive effects, schistosomula were treated with dsCYP450 RNA at a concentration that alone did not kill schistosomula (10 μg/mL) and miconazole at concentrations that resulted in minimal killing (2.5 or 5 μM) or each alone. Schistosomula cultures were set up as described above. A control experiment was set up with irrelevant dsRNA with and without 5 μM miconazole. Parasites were observed as described above.

### Total RNA isolation and cDNA synthesis

Total RNA was isolated from frozen worm and egg samples using the TRIzol Reagent (Life Technologies) per the manufacturer’s recommendation in a 2 ml Lysis Matrix Tubes (MP Biomedicals) containing 500 μl TRIzol reagent. Tubes were shaken three times for 20 seconds each using a tissue homogenizer (FastPrep-24 5G Instrument, MP Biomedicals). The samples were incubated on ice for 5 minutes in between each lysis process. After lysis, another 500 μl TRIzol Reagent was added to each sample, mixed and incubated at room temperature for 5 min. The resultant sample was spun at 13,000 x g for 1 min to pellet cellular debris. Following centrifugation, supernatants were transferred to a new 1.5 ml microfuge tube and extracted with chloroform/isopropanol according to the manufacturer’s instructions. The gelatinous, white RNA precipitate obtained after the chloroform/isopropanol extraction was resuspended in DEPC treated water in 75% ethanol and spun at 6500 x g for 5 min at 4°C. After centrifugation the supernatant was removed and the RNA pellet briefly air-dried and re-suspended in DEPC-treated water, heated briefly at 55°C quantified on a Nanodrop spectrophotometer. Total RNA was used for cDNA synthesis (iScript, BIO-RAD) per the manufacturer’s recommendation. The synthesized cDNA for each sample was quantified by Nanodrop spectrophotometry and stored at -20°C.

### Quantitative RT-PCR (qPCR) and semi-quantitative RT-PCR

Primers used for qPCR are shown in Table 2. α–tubulin (GenBank accession M80214) was used to normalize the results. The reactions were each carried out in a 20 μl reaction using ROX Passive Reference Dye (Bio-Rad) according to the manufacturer’s protocol. The amplification was monitored in a 7900HT Fast Real-Time PCR Machine (Applied Biosystems) under the following cycle conditions: (stage 1, 95°C 30 sec, stage 2, 95°C 5 sec, 60 C 30 sec) x 50, plus a one cycle dissociation curve. Fold differences were calculated using the 2^-ΔΔCT^ as described [33] with α–tubulin transcript levels serving as the internal standard. Reactions were done in triplicate. Semi-quantitative RT-PCR was used to assess the relative abundance of *Sm*CYP450 mRNA after RNAi silencing using Platinum Taq DNA polymerase (Life Technologies. Glyceraldehyde 3-phosphate dehydrogenase (GenBank accession M92359) was used as a control gene (primers GAPDH_S.mansoni FWD and GAPDH_S.mansoni REV) and *Sm*CYP450 cDNA was amplified with primers CYP450-interF2 and ORF_CYP450 Reverse.

## Results

### The *Sm*CYP450 coding sequence is similar to CYP450 proteins in other organisms

**Fig 2:**
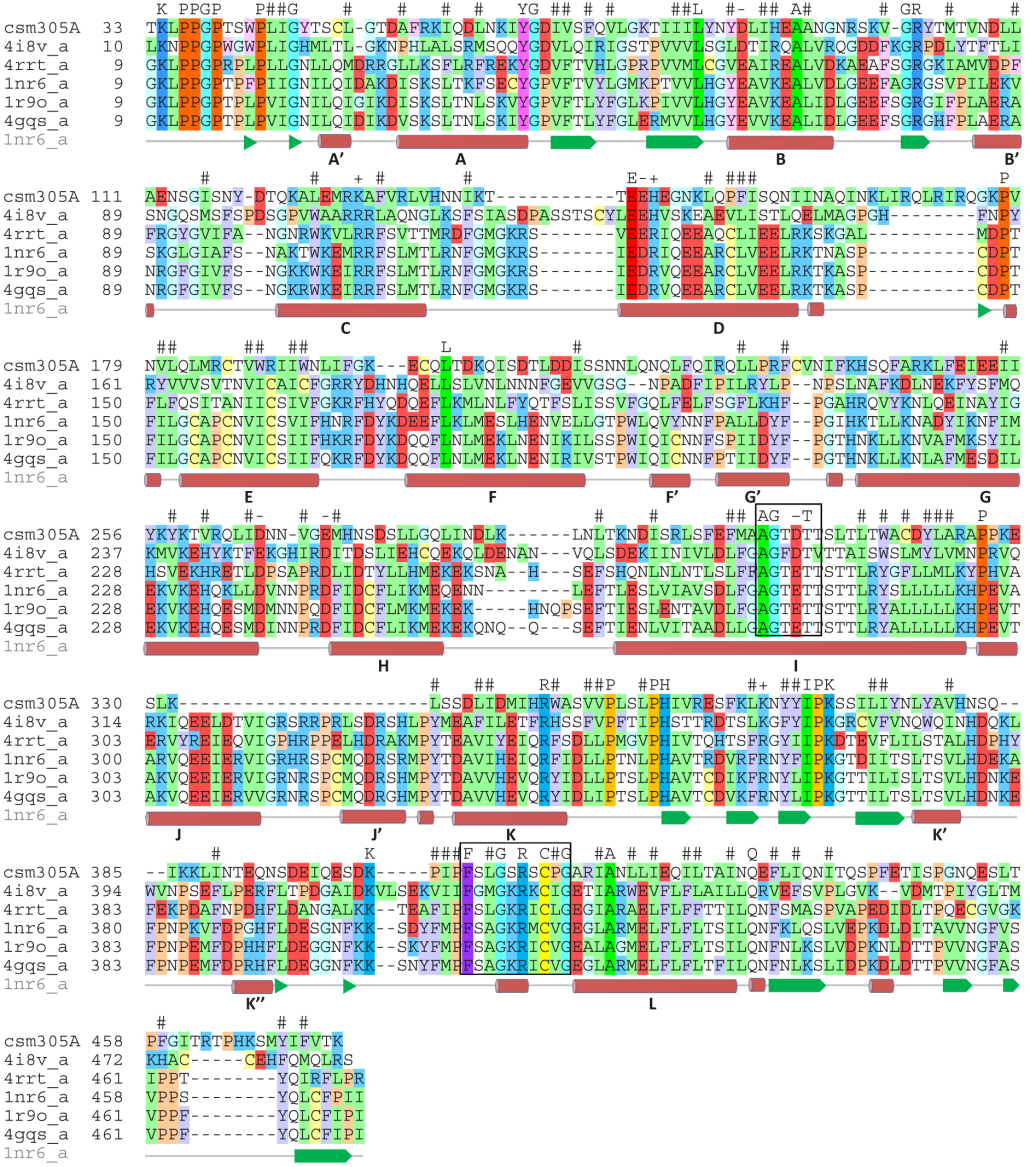
Comparison of *Schistosoma mansoni* CYP450 protein (Sman) with CYP450 proteins from other species. Multiple alignment of CYP450 proteins from *S*. *mansoni* (csm305A); rabbit CYP450 2C5 (1nr6_a); human CYP450 2C9 (1r9o_a); human CYP450 2C19 (4gqs_a); human CYP450 1A1 (4i8v_a); and human CYP450 2b6 (4rrt_a). The residues are shown in one letter code and colored by type: red- negatively charged, blue—positively charged, yellow—Cys, green—hydrophobic, cyan—Gly, ochre—Pro, purple—aromatic. The residues are shown in brighter colors for conserved positions. The ‘P450-signature’ sequence, which forms a channel for electron transfer, and the CYP450 consensus motif responsible for heme-binding and interaction with molecular oxygen and the relevant substrates are boxed. Predicted helices in the secondary structure based on homology modelling of SmCYP450 are indicated by the bold letters A-L based on rabbit CYP450 2C5 [38].

**Fig 3:**
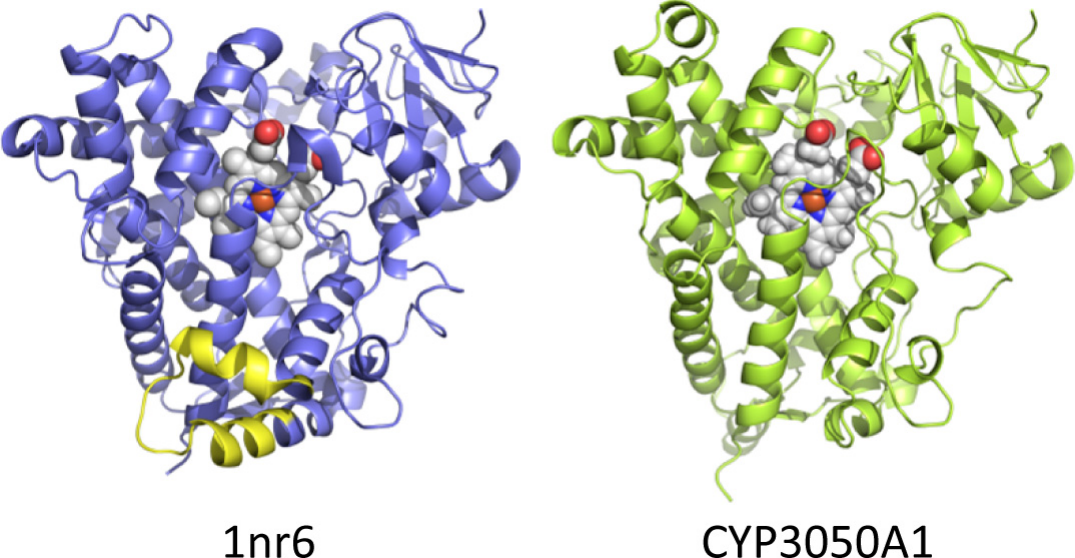
Structural modeling of *S*. *mansoni* CYP450 (CYP3050A1) and comparison to the structure determined for rabbit CYP450 2C5 (1nr6_a) [38]. The heme is shown is each model as a space-filling projection. The J and J’ helices in rabbit CYP450 2C5, which are absent in *S*. *mansoni* CYP450, are highlighted in yellow.

Cloning and sequence analysis shows that the *Sm*CYP450 coding sequence is 1452 base pairs encoding a protein of 483 amino acids with a predicted molecular weight of 55.28 kDa. The family assignment as CYP3050A1 was made by Dr. David R. Nelson according to the CYP450 nomenclature [34,35]. The sequence was found to be longer than the sequence reported in GeneBank (Smp_156400, 1245 base pairs,) due to a miscalled junction of the 5^th^ intron/6^th^ exon during genome annotation. The sequence obtained was submitted to GenBank with the accession number KT072747. The gene is composed of 7 exons and 6 introns spanning 15,378 base pairs (not including 5’ and 3’ noncoding sequences). Sequence analysis shows it to be comparable to CYP450 proteins from other organisms. The signature heme-binding motif [14,36], [**F**W]-[**S**GNH]-x-[**G**D]-{F}-[**R**KHPT]-{P}-C-[LIVMFA**P**]-[**G**AD], is present (the bold, underlined residues are present in *Sm*CYP450) (Fig 2). The ‘P450-signature’ sequence, [**A**G]-G-X-[**D**E]-T-[**T**S], which forms a channel for electron transfer [36], is also present in the *Sm*CYP450 peptide. The protein has an N-terminal membrane spanning region followed by the poly-proline domain, which is important for protein folding and structural integrity [37]. The turns by the poly-proline region provide a junction between the transmembrane region and the main catalytic domain typical for most CYP450 proteins [37]. The organization of the predicted secondary structure of the *Sm*CYP450 protein sequence follows other CYP450 proteins, beginning from helix A in the N-terminal region of the protein sequence and ending with helix L, which contains the heme-binding sequence (Fig 2). Likewise, with the exception of the absence of the J and J’ helices, the tertiary structure of *Sm*CYP450 protein is predicted to be similar to known CYP450 proteins (Fig 3).

### There is a single CYP450 protein in *S*. *mansoni*

It is possible that a diversity of CYP450 proteins or alternative subcellular targeting of *Sm*CYP450 results from alternative splicing or post-transcriptional modifications of the mRNA produced from the single *S*. *mansoni* CYP450 gene. This was addressed by analyzing cDNAs from a variety of developmental stages and by using modified 5’RACE and PCR with the schistosome spliced leader sequence to search for multiplicity of CYP450 mRNAs. We analyzed 33 clones from adult female worm cDNA, 23 clones from adult male worm cDNA, 11 clones from egg cDNA, and 28 clones generated by 5’ RACE and all sequences were identical. Therefore, we found no evidence for alternative splicing or other sequence variations. PCR with the spliced leader sequence and two different internal CYP450-specific primers resulted in no PCR products; therefore, the SmCYP450 mRNA does not appear to be trans-spliced. Therefore, it appears that the *Sm*CYP450 gene encodes a single CYP450 protein.

### *S*. *mansoni* CYP450 is differentially expressed during parasite development in the mammalian host

**Fig 4:**
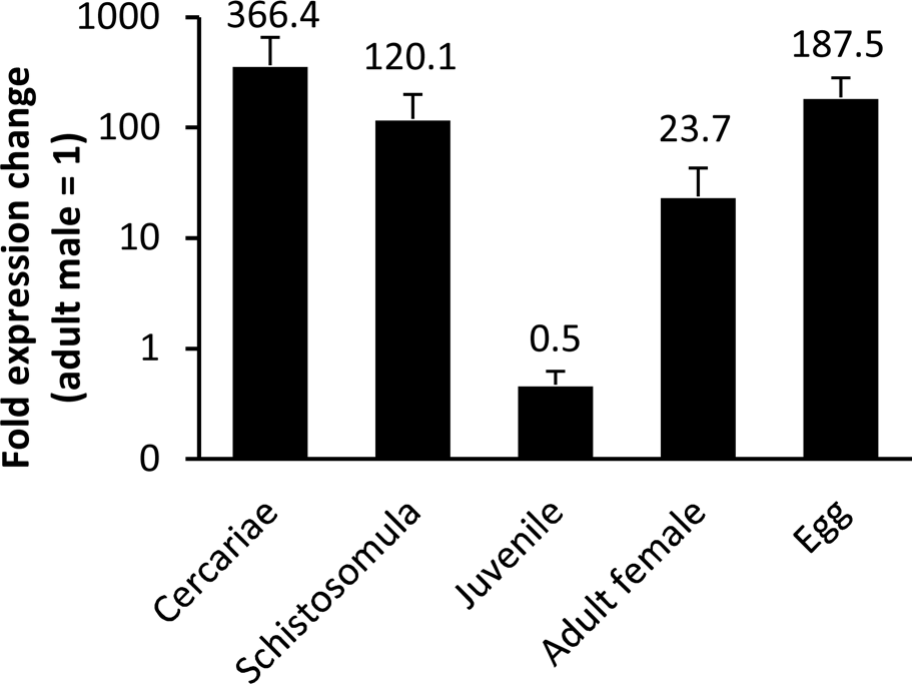
CYP450 messenger RNA abundance during the lifecycle of *Schistosoma mansoni*. Whole RNA was extracted from different stages of *S*. *mansoni* (cercariae, 1-day old schistosomula; juvenile liver worms (23 days post infection), adult males (49 days post infection), adult females (49 days post infection) and eggs) using TRIzol reagent and chloroform/ethanol extraction protocol. cDNA was synthesized from whole RNA and used for qRT-PCR, with reactions done in triplicate. Adult males (= 1) were used as calibrator stage and mRNA abundance was normalized to α-tubulin. Error bars indicate standard error of the mean with n ≥ 3 biological replicates. Numbers indicate fold change relative to adult males and all values are significantly different from adult males; p < 0.05; student t-test. The results indicate that *S*. *mansoni* CYP450 is expressed in all stages investigated and that its expression is developmentally regulated.

Using qRT-PCR we found that *Sm*CYP450 mRNA was present at all developmental stages investigated and that it is differentially present during development (Fig 4). Eggs, the larval stages of development (cercariae and schistosomula) and adult female worms had higher mRNA levels than adult male worms. Liver stage parasites had the lowest *Sm*CYP450 mRNA expression levels, about 50% that of adult males.

### *S*. *mansoni* CYP450 dsRNA treatment leads to schistosomula death

**Fig 5:**
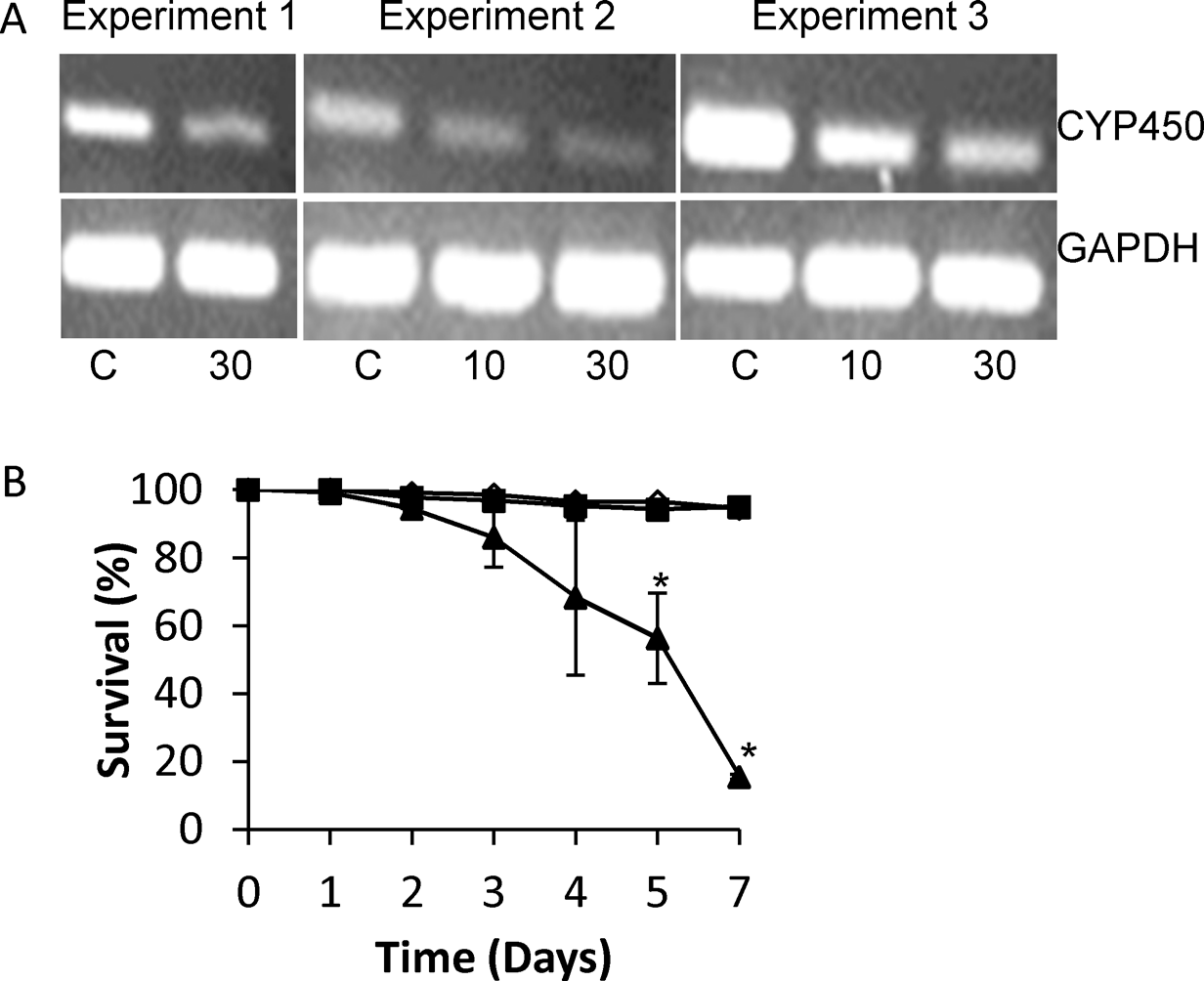
Effect of silencing *Schistosoma mansoni* CYP450 in cultured larval worms. Freshly prepared schistosomula (300–400) were placed in each well containing 1 ml Basch’s Media in a 24-well plate and overnight in a 37°C with 5% CO2. The following day schistosomula were treated with 10 or 30 μg/ml *S*. *mansoni* CYP450 dsRNA or 30 μg/ml negative control dsRNA. Over several days worms were observed for dead (dark, granular appearance) or alive (translucent). (A) mRNA expression patterns in schistosomula treated with *S*. *mansoni* CYP450 specific dsRNA or negative control dsRNA control after 3 days of treatment (Experiments 1 and 2) or 2 days treatment (Experiment 3). The control gene for cDNA input is *S*. *mansoni* glyceraldehyde 3-phosphate dehydrogenase (GAPDH). C, schistosomula treated with 30 μg/mL irrelevant dsRNA; 10, schistosomula treated with 10 μg/mL *Sm*CYP450 dsRNA; 30, schistosomula treated with 30 μg/mL *Sm*CYP450 dsRNA. (B) Effect of *S*. *mansoni* CYP450 dsRNA on schistosomula survival in cultures with 30 μg/mL negative control dsRNA (black square), 10 μg/mL *S*. *mansoni* CYP450-specific ds RNA (open triangle), and 30 μg/mL *S*. *mansoni* CYP450-specific ds RNA (black triangle). Treatments were done in triplicate and repeated 3 times. Error bars indicate standard error of the mean; *, p < 0.05; student t-test.

To determine if *Sm*CYP450 is essential for schistosomula survival we used RNAi to silence *Sm*CYP450 expression. Treating worms with 10 μg/mL or 30 μg/mL *Sm*CYP450 specific dsRNA for two or three days resulted in a dose-dependent reduction in *Sm*CYP450 message (Fig 5A). No change was seen in *Sm*CYP450 mRNA after treatment with 30 μg/mL irrelevant dsRNA or in GAPDH mRNA abundance after treatment with either dsRNA (Fig 5A). Treatment with 30 μg/mL *Sm*CYP450 specific dsRNA resulted in 80% schistosomula survival by day 3, 40% survival by day 5, and 15% survival by day 7. In contrast, 95% and 94.5% of schistosomula were alive on day 7 after treatment with 30 μg/mL irrelevant dsRNA or 10 μg/mL *Sm*CYP450 specific dsRNA, respectively (Fig 5B).

### The imidazole subgroup of azole antifungal CYP450 inhibitors is active against *S*. *mansoni*

**Fig 6:**
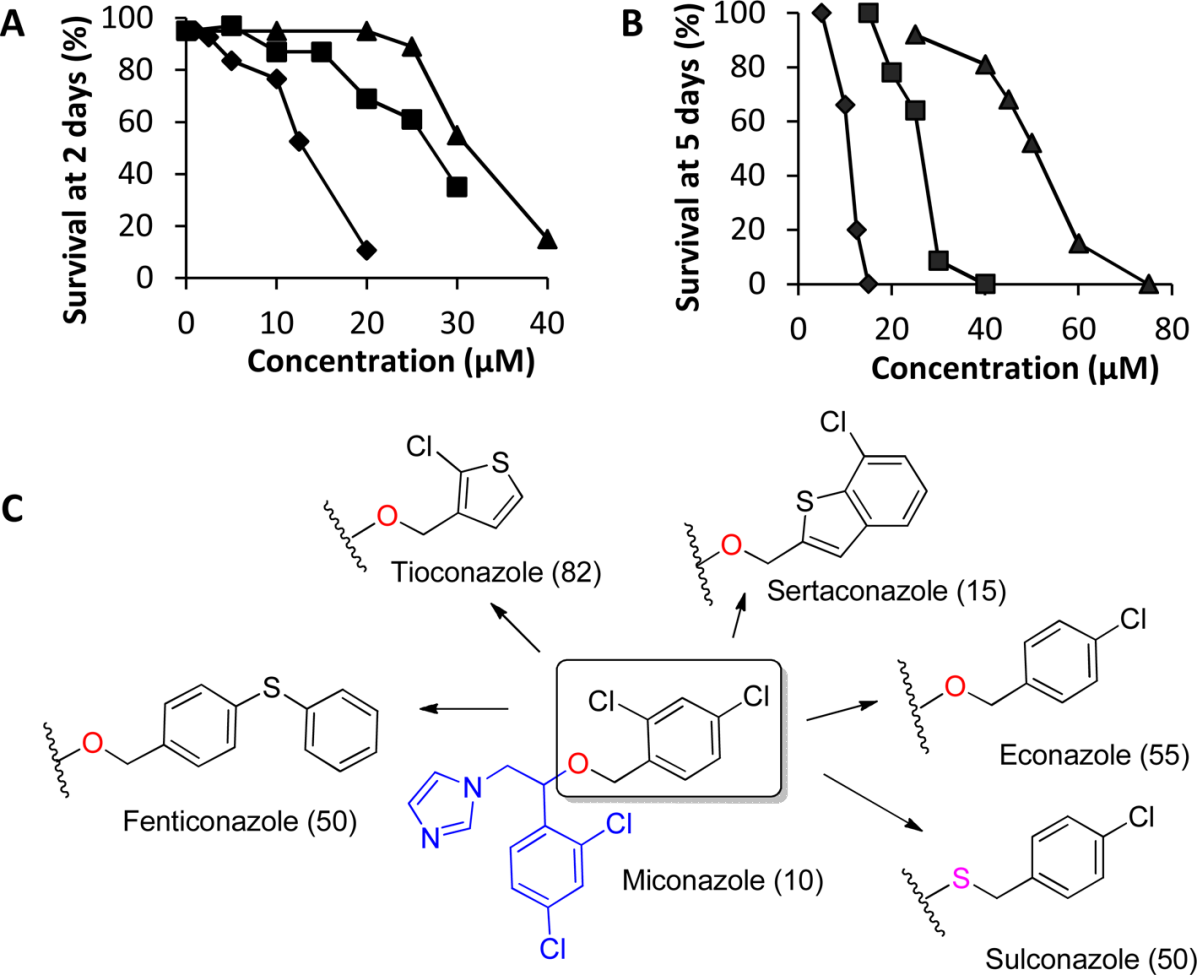
Activity of anti-fungal imidazole CYP450 inhibitors on larval and adult *Schistosoma mansoni* worms. Survival of schistosomula (A) after 2 d culture and adult worms (B) after 5 d culture for miconazole (black diamond), clotrimazole (black square), and ketoconazole (black triangle). (C) In house SAR on known miconazole analogs against adult worms. Numbers in the parenthesis are survival (%) of adult worms on day 7 in 10 μM of respective compound.

CYP450 enzymes are inhibited by numerous anti-infective and anticancer agents. We next asked if clinically relevant CYP450 inhibitors (Fig 1) affected parasite survival. Several antifungal imidazoles (miconazole, clotrimazole, ketoconazole) but not closely related triazole antifungals (fluconazole, posaconazole and triadimenol) were active against both larval and adult worms (Fig 6A and 6B and Table 3). Miconazole, clotrimazole, and ketoconazole had ED_50_ (Effective Dose producing 50% worm death) values of 10 μM, 20 μM, and 40 μM, after 5 day treatments against adult worms and 12.5 μM, 27.5 μM, and 30 μM after 2 day treatments against schistosomula, respectively. Other CYP450 inhibitors, such as prochloraz, sulfaphenazole, piperonyl butoxide, dafadine, letrozole, aminoglutethimide, abiraterone acetate, and anastrozole had no significant schistosomicidal activity against either larval or adult worms (Table 3). Expansion of the anti-fungal imidazole series was done to generate preliminary structure activity relationships of this compound series. Our studies revealed that imidazoles that retained the 1-(2,4-dichlorophenyl)-2-(1H-imidazol-1-yl)ethan-1-ol moiety of miconazole had significant schistosomicidal activity against both larval and adult worms, while those which lacked this moiety had much reduced or no schistosomicidal activity (Table 3, Fig 6C).

**Table 3:** Results with selected cytochrome P450 inhibitors used in this study.

Entry	Compound	Survival at 7 days (%)				Function and CYP450 class inhibited
		Adult		Schistosomula		
		5 μM	10 μM	5 μM	10 μM	
1	Miconazole	57	10	72	22	antifungal CYP51
2	Sertaconazole	95	15	65	6	
3	Sulconazole	100	50	90	56	
4	Fenticonazole	75	50	92	79	
5	Econazole	100	55	87	66	
6	Oxiconazole	100	60	89	71	
7	Tioconazole	100	85	86	82	
8	Bifoconazole	100	100	80	79	
9	Butoconazole	100	80	77	60	
10	Clotrimazole	n.d.^1^	100	97	90	
11	Ketoconazole	n.d.	100	98	97	
12	Fluconazole	100	100	98	94	
13	Posaconazole	100	100	94	88	
14	Triadimenol	100	100	99	99	
15	Prochloraz	100	100	94	82	
16	Sulfaphenazole	100	100	99	93	antibacterial CYP2C9
17	Piperonyl butoxide	100	100	100	100	pesticide CYP6D1
18	Abiraterone acetate	100	100	100	100	prostate cancer CYP17A1
19	Anastrozole	100	100	100	100	breast cancer CYP19A1
20	Letrozole	100	100	100	100	
21	Aminoglutethimide	100	100	100	100	
22	Dafadine	100	100	100	100	CYP27A1

^1^n.d., not determined.

### Miconazole targets *Sm*CYP450

**Fig 7:**
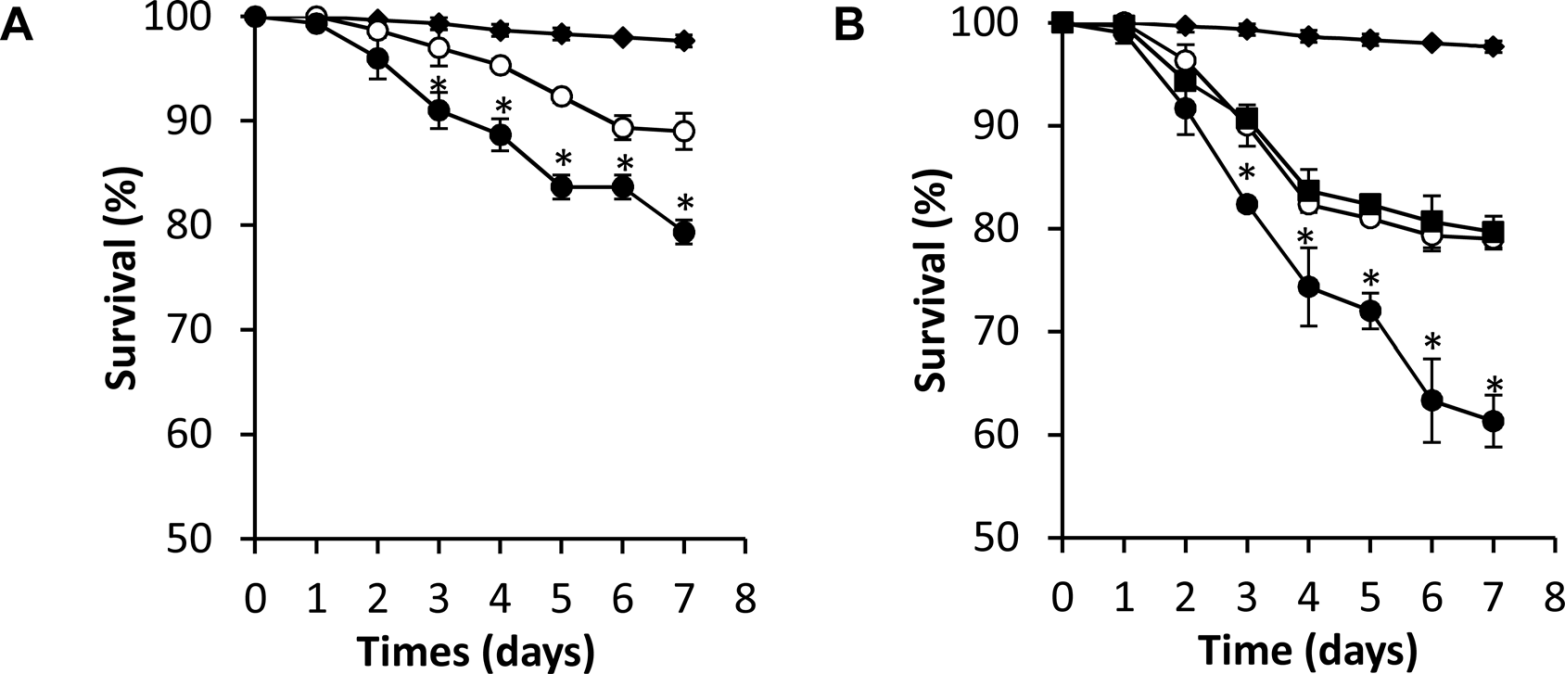
Combinations of miconazole and RNAi have increased killing activity, suggesting that they function through inhibition of the same target. (A) Schistosomula cultured with 10 μg/ml *S*. *mansoni* CYP450 dsRNA (black diamond); 2.5 μM miconazole (open circle); or 10 μg/ml *S*. *mansoni* CYP450 dsRNA and 2.5 μM miconazole (black circle). (B) Schistosomula cultured with 10 μg/ml *S*. *mansoni* CYP450 dsRNA (black diamond); 5 μM miconazole (black square); 5 μM miconazole plus 30 μg/ml irrelevant dsRNA and 5 μM miconazole (open circle); or 5 μM miconazole plus 10 μg/ml *S*. *mansoni* CYP450 dsRNA and 5 μM miconazole (black circle). All experiments were done in triplicate. Error bars indicate standard error of mean; *, p < 0.05; student t-test).

Does the potent schistosomicidal activity of miconazole act through inhibition of worm CYP450 or does it have other targets in the worm? To address this question we tested low doses of miconazole against worms treated with 10 μg/mL dsRNA CYP450, which caused no significant worm death itself. While 5 μM miconazole alone resulted in 80% survival after 6 days, combinations of 5 μM miconazole and 10 μg/mL SmCYP450-specific dsRNA resulted in 60% survival (p = 0.0042). Combining 2.5 μM miconazole (90% survival alone) and 10 μg/mL *Sm*CYP450-specific dsRNA resulted in 75% survival (p = 0.007) (Fig 7A). Addition of 30 μg/mL irrelevant dsRNA treatment had no effect on killing by 5 μM miconazole (Fig 7B). These results strongly suggest that miconazole schistosomicidal activity is specific for *Sm*CYP450.

### Miconazole treatment results in impaired schistosome egg development

**Fig 8:**
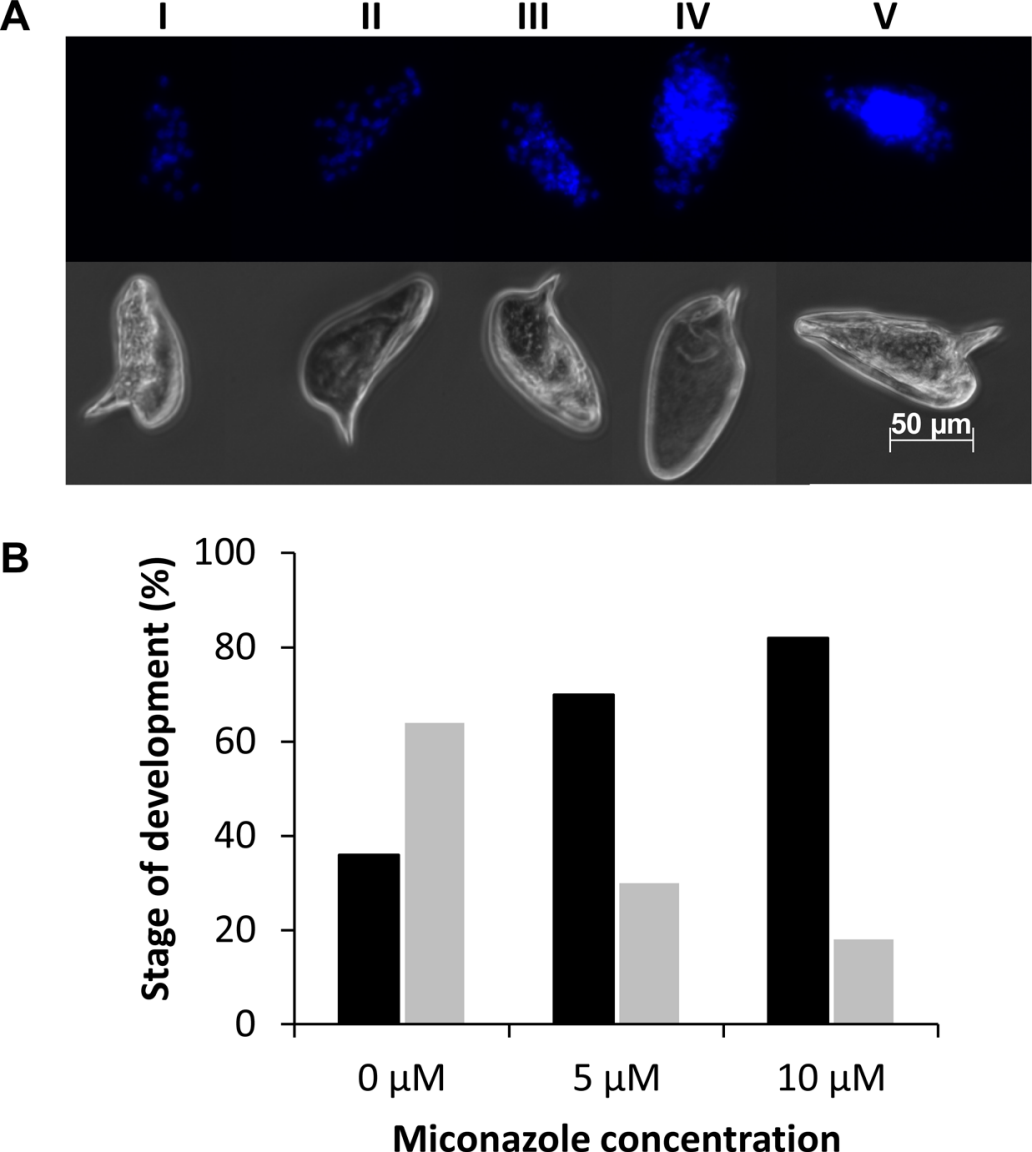
Effect of miconazole on egg development. (A) Example of egg development scoring scheme. Upper panel shows fluorescent images of eggs representative of each developmental stage scored; the bottom panel shows brightfield images of the same eggs. (B) Scoring of egg development in cultured eggs treated with 0, 5, or 10 μM miconazole. The percentage of eggs scored at developmental stages I-III (black bars) and eggs scored at developmental stages IV-V (gray bars) are indicated. For 0 μM miconazole, n = 55 eggs scored; for 5 μM miconazole, n = 56 eggs scored; for 10 μM miconazole, n = 62 eggs scored.

To determine if miconazole interferes with egg development and maturation we treated eggs deposited by freshly perfused adult worm pairs with miconazole and monitored embryo development using a recently described method [32,39]. Egg development was scored based on the number and arrangement of cell nuclei (Fig 8A). Our results indicate that there is a general interference of egg development and accumulation of early embryonic stages (I, II and III) and decrease in late stage embryos (IV and V) in the miconazole treatments compared to the DMSO controls. Only 30% (18/62) of eggs treated with 5 μM miconazole and 18% (10/56) treated with 10 μM miconazole reached the latter stages of egg development (stages IV and V) compared to 64% (35/55) in DMSO control (Fig 8B). These results indicate that miconazole affects embryonic development.

## Discussion

Because schistosomiasis control relies on a single drug and there is field evidence for the evolution of drug resistance [3,4], there is an urgent need to identify new, druggable worm targets. In this study we present the first detailed characterization of the CYP450 from *S*. *mansoni* and provide strong evidence that it is an essential and druggable target in the worm.

The *Sm*CYP450 exists as a single copy gene in the *S*. *mansoni* genome [16]. This is in stark contrast to humans, which have 57 genes and alternative splicing and genetic variations that can lead to the production of many more distinct protein species [40,41], and to the free-living flatworm *Schmidtea mediterranea*, which has at least 39 CYP450 genes [15]. The loss of CYP450 family members in parasitic helminths has been noted previously [42]. However, the fact that parasitic flatworms have retained one CYP450 signifies that it plays an important and perhaps essential function. We add here that in *S*. *mansoni* there appears to be no post-transcriptional modifications (alternative or trans-splicing, RNA editing) to the mRNA. Therefore, it is likely that a single protein product is produced from the *Sm*CYP450 gene. Since there was no evidence for alternative splicing to insert different leader sequences at the N-terminus, the protein product is likely only targeted to the endoplasmic reticulum.

The predicted protein has generally low sequence identity with the other CYP450s; the highest identity to human CYP450 proteins is 22% to CYP2C9. Importantly, the CYP450 consensus motif responsible for heme-binding and interaction with molecular oxygen and the relevant substrates and the ‘P450-signature’ sequence are conserved in the *Sm*CYP450 protein sequence. Curiously, *Sm*CYP450 lacks a number of motifs found in many characterized CYP450. The majority of CYP450s contain an ‘EXXR motif’ in helix K. The glutamic acid and arginine residues form a charge pair with a third amino acid more distant in the meander region. This is frequently an arginine in the so-called ‘PERF motif’. Putative functions of the EXXR motif and PERF motif may be to associate heme with the newly synthesized CYP450 polypeptide and/or to maintain the CYP450 tertiary architecture [43]. This is key to the structural fold of CYP450s and previous studies in which mutagenesis directed at the side-chains of glutamic acid or arginine in the EXXR motif or at the invariant cysteine in the L-helix resulted in completely inactive and misfolded proteins [44]. However, these motifs are not present in CYP450s from parasitic Trematodes (*e*.*g*., *Schistosoma*, *Clonorchis sinensis*) and Cestodes (*e*.*g*., *Echinococcus multilocularis*) [42]. Their absence is not without precedent as the EXXR motif is also absent in most members of a CYP157 subfamily in *Streptomyces* spp [45]. The Trematode CYP450 proteins also lack the J and J’ helices, which occur to the N-terminal side of and include the EXXR motif. How these differences affect protein structure and function remains to be determined.

CYP450s function in an electron transport chain in which electrons are passed from NADPH through a flavoenzyme either directly to the CYP450 heme or indirectly through cytochrome *b*5 or ferredoxin. In the endoplasmic reticulum, the flavoenzyme is NADPH CPY450 reductase. Additional partners of CYP450s in the endoplasmic reticulum include cytochrome *b*5 and cytochrome *b*5 reductase. In mammals, ferredoxin reductase and ferredoxins (also known as adrenodoxin reductase and adrenodoxins) are found in the mitochondria and are involved in steroid hormone synthesis mediated by CYP450s. The *S*. *mansoni* genome contains one CYP450 reductase, two cytochrome *b*5s, two cytochrome *b*5 reductases, one ferredoxin reductase, and two ferredoxins with potential to support *Sm*CYP450 activity. Previous studies in schistosomes have found that ferredoxin reductase is mitochondrial and likely functions there in redox defenses [46,47]. Since we currently have no evidence for mitochondrial targeting of *Sm*CYP450 protein, it is not likely that it functions in concert with ferredoxin reductase/ferredoxins.

Unlike the only previously characterized trematode CYP450, which showed highest expression in adult hermaphrodites [42], *Sm*CYP450 is expressed at the highest levels in larval and egg stages. It is important to note that the developmental cycles and tissue locations of these organisms are significantly different. After active host localization and penetration, *S*. *mansoni* has extensive interactions with host skin, lungs, liver, and vascular epithelia, while *Opisthorchis* worms reside in the bilary ducts after excysting from metacercariae in the duodenum. As sequence identity between *S*. *mansoni* and *O*. *felineus* CYP450 proteins is only 37% it is quite possible that CYP450s have different functions in the worms.

The function of the *Sm*CYP450 is not yet known. Different development stages may require different CYP450 metabolites and/or experience different immunological stresses. For instance larval parasites penetrate the skin of human host and begin migration through the skin and other tissue and may encounter different stress and immunological responses than adult worms in the mesenteric system. Larval schistosomes synthesize and secrete eicosanoids [48–53], which are signaling molecules derived from arachidonic acid, some of which are produced by CYP450s. The eicosanoids produced by schistosomes may down modulate host immune function [54,55]. Eicosanoids produced by adult worms may control other functions such as vasodilator activity, and/or vasoconstrictive action [55].

Other potential functions of *Sm*CYP450 are in the metabolism of cholesterol and steroid hormones. Adult worms have been shown to convert cholesterol into several metabolites including pregnenolone, the first committed metabolite in steroid hormone biosynthesis [56,57]. Male worms transfer cholesterol and uncharacterized cholesterol metabolites to female worms [56] and synthetic steroids have been shown to affect worm egg production *in vivo* [56]. More recently, a catechol-estrogen conjugate (downstream products of CYP450 metabolism of estradiol and estrone), which has anti-estrogen affects, was identified in schistosome worm extracts and in the serum of infected humans [58]. Retinoic acid is essential for embryonic development in all metazoan organisms investigated, including free-living flatworms [59]. Retinoic acid activity is controlled through its tightly regulated synthesis from vitamin A (all-trans retinol) in a 2-step process by retinol dehydrogenases to all-trans retinal and by retinaldehyde dehydrogenases to all-trans-retinoic acid and is terminated via its breakdown by CYP450s [18,60]. Although retinoic acid signaling or metabolism in schistosomes is largely unknown, they have enzymes involved in retinoic acid metabolism (10 retinol dehydrogenases and 2 retinaldehyde dehydrogenases) and nuclear receptors related to retinoic acid receptors [61–64]. Ecdysteroids are hormones involved in insect molting and development and CYP450s are involved in their synthesis and transformation from farnesyl diphosphate and cholesterol. Ecdysteroids have been detected in schistosomes and their levels shown to vary during development [65,66]. *S*. *mansoni* synthesizes ecdysone and 20-OH ecdysone, which were shown to be potent stimulators of growth and vitellogenesis [67]. β-Ecdysterone was found to be effective in stimulating host location activities in *S*. *mansoni* miracidia [68]. Worms have two nuclear receptors related to insect ecdysone receptors, but their function in ecdysteroid signaling has not been determined [69,70]. Identification of the function of *Sm*CYP450 will be targeted in future studies.

Our findings indicate *S*. *mansoni* has a single CYP450 protein, with highest sequence identity to human CYP450s CYP2C9 and CYP1A1. In order to compare the differences between *S*. *mansoni* CYP450 and human CYP450s we tested several different classes of CYP450 inhibitors. Although miconazole and structurally related imidazoles had schistosomicidal activity against adult and larval worms, other CYP450 inhibitors did not. These observations gave rise to an early exploration to investigate the structure-activity-relationships (SAR) of imidazole class of compounds, especially miconazole analogs (Fig 6C). Miconazole analogs were obtained by substituting the (2,4-dichlorophenyl)methanol moiety with different aryl groups. Sertaconazole, which results from substitution with a (7-chlorobenzo[b]thiophen-2-yl) methanol group, was equipotent to miconazole against adult and larval worms. Replacement with (4-chlorophenyl) methanol group results in econazole. Replacement of the oxygen by a sulfur in the econazole led to sulconazole. Modification of the econazole by substitution of a phenylthio group for the 4-chloro led to fenticonazole. Replacement with an oxime moiety into the miconazole gave oxiconazole. Econazole, sulconazole, fenticonazole and oxiconazole were less potent than miconazole. Substitution with (2-chlorothiophen-3-yl) methanol moiety results tioconazole, which is much less active. Our results indicate that miconazole constitutes a promising scaffold for targeting schistosome worms. Evidence that schistosomicidal activity of miconazole and analogs resides in the 1-(2,4-dichlorophenyl)-2-(1H-imidazol-1-yl)ethan-1-ol moiety of miconazole suggests routes to improved activity by rational drug design in future studies.

Miconazole had previously been included in a medium throughput phenotypic screen against schistosomula in an effort to repurpose approved drugs [71]. In, this study, compounds were screened at 1 μM against schistosomula and miconazole was found to be inactive, which is consistent with our results. However, for our screening purposes we tested compounds at higher concentrations and therefore, identified the schistosomicidal activity of this class of compounds. Although the concentrations required for worm killing activity *in vitro* may not be attained *in vivo* due to low biological availability, improved pharmacological properties can be incorporated into miconazole analogs to overcome these limitations. Our results indicate that the schistosomicidal activity of miconazole is due to inhibition of *Sm*CYP450. Low concentrations of miconazole alone resulted in low schistosomicidal activity and partial reduction of *Sm*CYP450 mRNA alone resulted in no larval worm death. However, combination treatments produced more than an additive response: 10% death in 2.5 μM miconazole alone increased to 20% with partial mRNA silencing and 20% death in 5 μM miconazole alone increased to 40% with partial mRNA silencing. The simplest explanation for this effect is that partial mRNA silencing results in decreases in *Sm*CYP450 protein, which although it is not lethal to the worms itself, results in increased activity of miconazole due to a reduction in its protein target abundance. This strongly suggests that both *Sm*CYP450 dsRNA and miconazole target the same pathway.

In schistosomes, egg development is a multi-stage process. Within the host mesentery and vasculature, a mature female releases approximately 300 encapsulated embryos (pre-mature eggs) per day [72,73]. Prior to that and within the mature female the early development of eggs occurs in several pre-zygotic and post zygotic stages [74]. Using methods recently developed to facilitate monitoring egg development [32,38] we investigated the effect of miconazole on egg development and maturation. Treatment with miconazole resulted in a dose-dependent impairment of *ex vivo* egg development, with most miconazole-treated eggs remaining at the early stages of embryonic development (Stages I-III) compared to control treatments, in which most eggs reached later stages of embryonic development (stages IV and V). CYP450 proteins are known to be involved in egg development in *C*. *elegans*, with CYP31A2 and CYP31A3 essential for the production of lipids required for egg shell development [20]. In addition, retinoic acid is essential for embryonic development in all metazoan organisms investigated, including free-living flatworms, and as indicated above, retinoic acid metabolism is mediated by CYP450 proteins. Inhibition of retinoic metabolism by miconazole could interfere with embryogenesis and egg development. There has not been a direct identification of *Sm*CYP450 protein in eggs [75]. The newly oviposited egg is not fully formed and undergoes embryonic and subshell envelope development [76]. It is not known if *Sm*CYP450 functions in embryonic or subshell envelope development or both, but our work shows for the first time that miconazole can block egg development.

Schistosomiasis remains a challenging disease to people living in endemic areas. In spite of many years of praziquantel use, the prevalence of infection remains high. The specter of evolving resistance to praziquantel, the only drug available for disease treatment, calls for the identification of new protein targets, the discovery of lead compounds and the development of new drugs for the treatment of the disease. The *S*. *mansoni* CYP450 exists as a single gene in the parasite genome. Our work shows that it is essential for parasite survival and could be an ideal drug target. In addition, select anti-fungal azoles could be promising starting points for future studies towards identifying new therapies for schistosomiasis.
