# Correction: The *Schistosoma mansoni* Cytochrome P450 (CYP3050A1) Is Essential for Worm Survival and Egg Development

**DOI:** 10.1371/journal.pntd.0004439

**Published:** 2016-02-01

**Authors:** Peter D. Ziniel, Bhargava Karumudi, Andrew H. Barnard, Ethan M. S. Fisher, Gregory R. J. Thatcher, Larissa M. Podust, David L. Williams

The following information is missing from the Funding section: Research funding was provided by the Department of Immunology and Microbiology at Rush University. We acknowledge the Research Open Access Publishing (ROAAP) Fund of the University of Illinois at Chicago for financial support towards the open access publishing fee for this article.
